# RETRACTION: Systems Pharmacology Reveals the Mechanism of Activity of Physalis alkekengi L. var. Franchetii Against Lipopolysaccharide‐Induced Acute Lung Injury

**DOI:** 10.1111/jcmm.70950

**Published:** 2025-11-12

**RETRACTION**: Y. Yang, Z. Ding, Y. Wang, R. Zhong, Y. Feng, T. Xia, Y. Xie, B. Yang, X Sun. and Z. Shu , “Systems Pharmacology Reveals the Mechanism of Activity of Physalis alkekengi L. var. Franchetii Against Lipopolysaccharide‐Induced Acute Lung Injury,” Journal of Cellular and Molecular Medicine 24, no. 9 (2020): 5039–5056, 10.1111/jcmm.15126.32220053 PMC7205831

The above article, published online on 27 March 2020 in Wiley Online Library (wileyonlinelibrary.com), has been retracted by agreement between the authors; the journal Editor‐in‐Chief, Stefan N. Constantinescu; the Foundation for Cellular and Molecular Medicine, and John Wiley and Sons Ltd. The retraction has been agreed upon following an investigation into concerns raised by a third party. The investigation confirmed that western blot bands presented in Figures 4D and 5B were found to be duplicated in another article published earlier by a different group of authors. Furthermore, the duplicated bands were shown to represent different proteins. Although the authors cooperated with the investigation and were able to provide some new data from experiments conducted post publication, they were unable to provide the original blots or account for the duplication of the blots in a previous study. As a result, the editors consider the results and conclusions of this article to be invalid.

